# Fine‐scale ecological and anthropogenic variables predict the habitat use and detectability of sloth bears in the Churia habitat of east Nepal

**DOI:** 10.1002/ece3.8512

**Published:** 2022-01-13

**Authors:** Manoj Pokharel, Asmit Subba, Dipa Rai, Simrik Bhandari, Yadav Ghimirey

**Affiliations:** ^1^ Department of Environmental Science Tri‐Chandra Multiple Campus Kathmandu Nepal; ^2^ Central Department of Zoology Tribhuvan University Kathmandu Nepal; ^3^ Department of Environmental Science GoldenGate International College Kathmandu Nepal; ^4^ Department of Environmental Science and Engineering Kathmandu University Dhulikhel Nepal; ^5^ Friends of Nature Kathmandu Nepal

**Keywords:** Churia hills, detectability, habitat use, Nepal, occupancy, sloth bear

## Abstract

Once widespread throughout the tropical forests of the Indian Subcontinent, the sloth bears have suffered a rapid range collapse and local extirpations in the recent decades. A significant portion of their current distribution range is situated outside of the protected areas (PAs). These unprotected sloth bear populations are under tremendous human pressures, but little is known about the patterns and determinants of their occurrence in most of these regions. The situation is more prevalent in Nepal where virtually no systematic information is available for sloth bears living outside of the PAs. We undertook a spatially replicated sign survey‐based single‐season occupancy study intending to overcome this information gap for the sloth bear populations residing in the Trijuga forest of southeast Nepal. Sloth bear sign detection histories and field‐based covariates data were collected between 2 October and 3 December 2020 at the 74 randomly chosen 4‐km^2^ grid cells. From our results, the model‐averaged site use probability (*ψ* ± *SE*) was estimated to be 0.432 ± 0.039, which is a 13% increase from the naïve estimate (0.297) not accounting for imperfect detections of sloth bear signs. The presence of termite mound and the distance to the nearest water source were the most important variables affecting the habitat use probability of sloth bears. The average site‐level detectability (*p* ± *SE*) of sloth bear signs was estimated to be 0.195 ± 0.003 and was significantly determined by the index of human disturbances. We recommend considering the importance of fine‐scale ecological and anthropogenic factors in predicting the sloth bear‐habitat relationships across their range in the Churia habitat of Nepal, and more specifically in the unprotected areas.

## Graphical abstract

We undertook a spatially replicated sign survey‐based single‐season occupancy study intending to overcome the ecological information gap for one of the strongholds of sloth bear population residing in the Churia habitat located outside the protected areas of east Nepal.

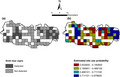

## 1 INTRODUCTION

Among the terrestrial mammals, large‐bodied species inhabiting the lowland areas of the developing regions are at greater risk of human‐induced extirpation (Schipper et al., 2008). One particular cause for this is the widespread habitat loss and degradation undergoing in these areas, limiting the ability of large mammals to meet their complex biological requirements (Cardillo et al., 2005; Ceballos & Ehrlich, 2002). For example, in the tropics of South and Southeast Asia, increased habitat conversion has isolated most of the threatened large mammals to generally small protected reserves, whereas remaining natural habitats outside the reserves are largely fragmented and degraded (Wikramanayake et al., 2004; Wong & Linkie, 2013). Population dispersal of certain charismatic species (e.g., tiger, elephant) has been facilitated through the initiation of landscape‐scale habitat connectivity approaches (Brodie et al., 2016). But, such single or few species‐focused management approaches often come at the cost of undermining the ecological needs and threats of many other sympatric species that have important ecological and conservation value (Wang et al., 2018, 2021). This is especially true for species having a less charismatic demeanor with poor representation in the network of PAs (Guan et al., 2015; Wang et al., 2021). The south Asian endemic sloth bear (*Melursus ursinus*) exemplifies the conservation challenges faced by such species (Puri et al., 2015).

Sloth bears in general are a lowland species that were once widespread throughout the tropical forests of the Indian subcontinent (Brander, 1982). However, over the past decades, they have suffered rapid range collapse and local extirpations, leading to patchy distributions in lowland habitat remnants of India, Nepal, Sri Lanka, and probably Bhutan (Garshelis et al., 1999; Yoganand et al., 2006). It has been estimated that more than half of the sloth bears’ remaining range is not under any forms of protection (Dharaiya et al., 2016). In these areas, sloth bears are under tremendous human pressures arising primarily from large scale habitat loss and degradation, and to a lesser extent from poaching and harvest of live cubs for use as “dancing bears” (D’Cruze et al., 2011; Dharaiya et al., 2016; Garshelis et al., 1999). Additionally, sloth bears are perceived as a dangerous species due to their frequent involvement in human attacks. As a result, locals support for conservation has eroded, and bears often become a subject of human persecution for retaliation or self‐defense (Debata et al., 2017; Garcia et al., 2016; Ratnayeke et al., 2014). Given the lack of enforcement in abating most of these threats in the unprotected regions, >30% of sloth bears’ population is projected to decline within the next few decades (Dharaiya et al., 2016).

Furthermore, the conservation of sloth bears is hindered by the lack of scientifically sound information required for effective conservation planning. There are rough estimations regarding the overall distribution and population status of sloth bears (Garshelis et al., 1999). Though studies examining human‐sloth bear conflict are emerging (Debata et al., 2017; Dhamorikar et al., 2017; Garcia et al., 2016; Prajapati et al., 2021; Ratnayeke et al., 2014; Sharp et al., 2020), research on sloth bear‐habitat relationships and space use patterns are very limited, mostly restricted to a few geographic landscapes of their entire range (Joshi et al., 1995; Puri et al., 2015; Ratnayeke et al., 2007; Srivathsa et al., 2018). Being a relatively widespread species with observed geographic variations in the use of resources and habitats (Joshi et al., 1995; Ratnayeke et al., 2007), understanding the fine‐scale patterns and drivers of sloth bear occurrence become crucial for effective site‐specific conservation planning. Such information would be especially vital in managing the populations residing in the fragmented landscapes outside of PAs (Akhtar et al., 2004; Puri et al., 2015).

Sloth bears in Nepal are a nationally endangered species (Jnawali et al., 2011). They have a small estimated population (<250 adults) and a narrow range of distribution in the fragmented forests of southern lowlands and adjacent Churia hills (Garshelis, Joshi and Smith, 1999; Jnawali et al., 2011). Four PAs provide formal protection to sloth bears in this range, but a large portion of their habitat remains unprotected (Garshelis et al., 1999). They are fairly common and somewhat comprehensively studied in the Chitwan National Park (CNP) of central Nepal, elsewhere they are considered rare with poor ecological information available (Garshelis et al., 1999; Garshelis, Joshi and Smith, 1999). Limited remnants of natural lowland habitats outside the PAs have made the Churia hills to be the last refuge for sloth bears in these areas (Garshelis et al., 1999). However, there is inadequate information about the sloth bears inhabiting the Churia hills. Even the baseline reports on the distribution and abundance are extrapolated based on the opinions of experts and locals (Garshelis et al., 1999; Jnawali et al., 2011). Efforts made to verify such reports and investigate the local‐level habitat correlates of sloth bears are extremely rare.

We carried out this study as an effort of bridging this information gap for sloth bears inhabiting the typical Churia habitat in the Trijuga forest of east Nepal. This forest is known to shelter one of the probable strongholds of the sloth bear populations (40–50 individuals) within Nepal. But, again the assessment is grounded on the anecdotal evidences provided by the locals (Jnawali et al., 2011). A few recent studies corroborated the presence of sloth bears and also revealed the issue of human‐sloth bear conflict in parts of this region (Pokharel & Aryal, 2020; Subedi et al., 2021). This has made it essential to investigate how the sloth bears use this forest patch, so that appropriate local‐level conservation and management plans can be devised. We used sign survey‐based single‐season occupancy modeling to reliably elucidate the patterns and determinants of habitat use by sloth bears in the Trijuga forest. The obtained findings provide baseline data with implications for the design of future studies targeted at sloth bears in the Trijuga forest as well as similar areas of the Churia hills.

## 2 MATERIALS AND METHODS

### 2.1 Study area

**FIGURE 1:**
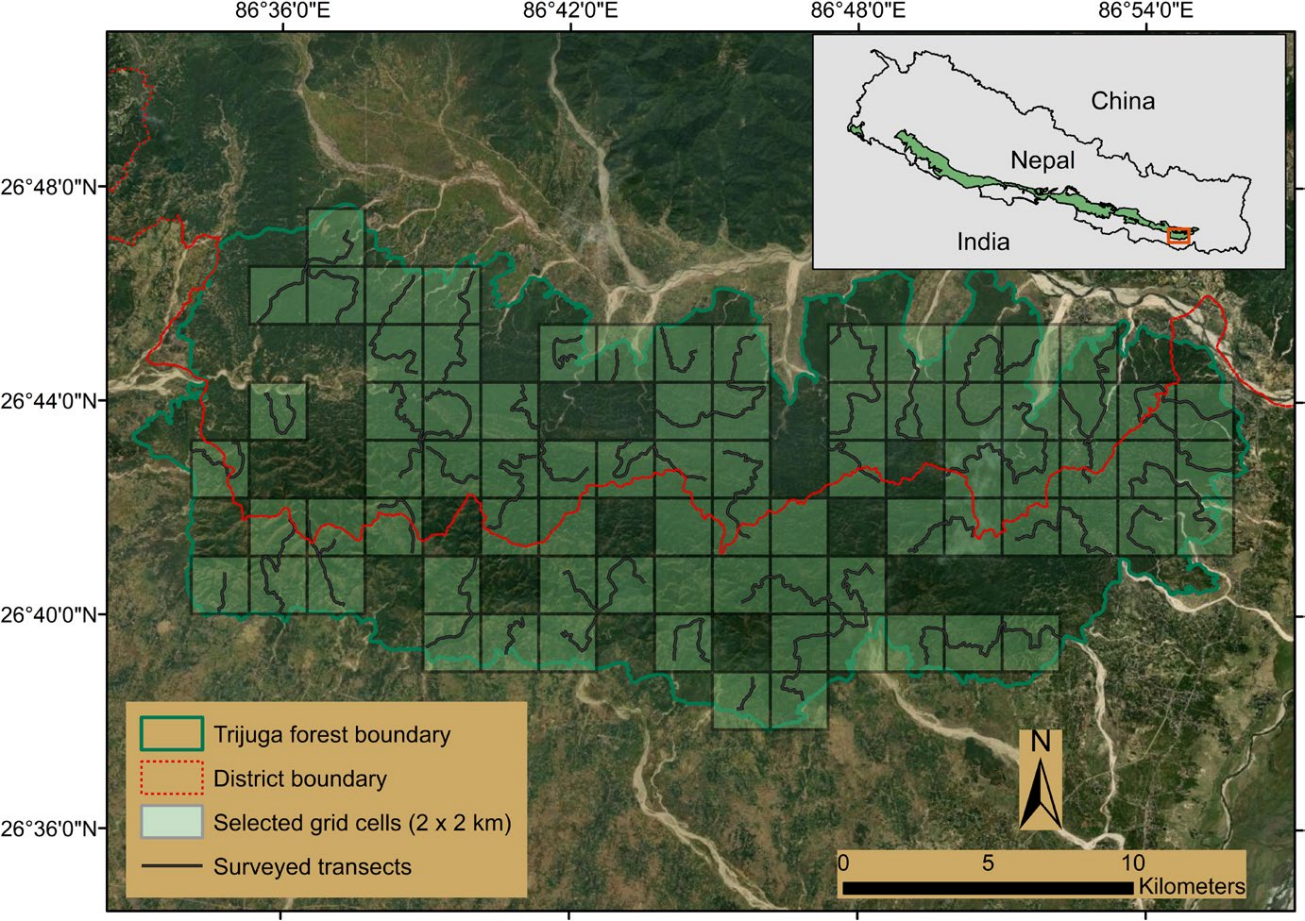
Map of Trijuga forest with the selected grid cells (2 × 2 km) and delineated transects for sign‐based occupancy surveys. Inset map shows the location of the study area in reference to the predicted distribution range of sloth bears in Nepal, adapted from IUCN Red List 2020

The Trijuga or Triyuga forest is one of the largest remaining patches of lowland forest outside the PAs of Nepal. It is approximately 430 km^2^ in size and is distributed under 9 municipalities of Udayapur and Saptari districts that fall under the administration of Province 1 and Province 2, respectively (Aryal et al., 2020). This forest is a part of the Churia hills that runs east to west parallel to the Himalayas in Nepal (Subedi et al., 2021). The peripheral areas of the forest are managed as community forests and the remaining is designated as national forest. Extensive agricultural lands interspersed with human settlements surround the Trijuga forest from all sides except the north‐western part where the habitat is connected through a narrow patch to the Churia range moving westward (Figure 1). The lower tropical ecological zone dominates this region with average annual temperature and precipitation falling in the range of 23–25.5°C and 1159–2827 mm, respectively (Lillesø et al., 2005). The elevation of the forest ranges from 104 to 430 m. Soil erosion, landslides, and flash floods are frequent in this area during the peak monsoon (June–September) similar to the other parts of the geologically fragile Churia hills (Ghimire et al., 2013). On the other hand, the summer season (March–June) is associated with extensive dryness leading to less availability of water sources and forest fires (Thapa & Kelly, 2017).

Vegetation of the Trijuga region consists of both dry as well as moist deciduous forests. *Shorea robusta* dominates much of the northern part of the forest. Progressing toward the south, the vegetation is slowly replaced by mixed deciduous forest, and it becomes the dominant forest type in the southern boundary. *Dalbergia latifolia*, *Acacia catechu*, *Terminalia tomentosa*, and *Semicarpous anacardium* are the commonly found trees in mixed deciduous forest. Deciduous riverine forest mostly dominated by *Dalbergia sisoo* and *Acacia catechu* is prevalent along the river banks of the study area. In addition to sloth bears, mammalian faunas, such as common leopard (*Panthera pardus*), Asiatic elephant (*Elephas maximus*), barking deer (*Muntiacus muntjak*), wild boar (*Sus scrofa*), jungle cat (*Felis chaus*), golden jackal (*Canis aureus*), Bengal fox (*Vulpes bengalensis*), rhesus macaque (*Macaca mulatta*), and Tarai gray langur (*Semnopithecus hector*), can be found in the Trijuga forest (Aryal et al., 2020; Shah et al., 2018). Local people of the area are highly dependent on the forest resources for their livelihood and they harvest products from different plants including those that are of dietary importance to sloth bears. Some of such shared plant resources are *Bombax ceiba*, *Ficus* sps., *Cassia fistula*, *Magnifera indica*, *Zizyphus* sps., *Aegle marmelos*, *Bridelia retusa*, *Syzygium cumini*, and *Phoenix humilis* (Shah et al., 2018).

### 2.2 Sampling design

We intended to analyze the occurrence probability of sloth bears at fine spatial scale, such that the estimates derived from occupancy modeling were interpreted as the probability of “site use” and not the probability of “site occupancy” (Mackenzie & Royle, 2005). We adopted a sampling framework that consisted of sample units as grid cells of 4 km^2^. The size of the sample unit is smaller than the known home range of sloth bears across much of their range (Joshi et al., 1995; Yoganand et al., 2005; *but see* Ratnayeke et al., 2007), and is considered adequate enough to study the fine‐scale spatial pattern, even for species with much larger home range than sloth bears (e.g., Kafley et al., 2016). We overlaid 144 4 km^2^ grid cells on the land cover map of Trijuga forest using the Fishnet tool in ArcGIS 10.4. We eliminated 35 grid cells that fell on the forest edges and had <50% area (<2 km^2^) within the forest boundary, after deciding them to be less suitable for use by sloth bears through direct habitat observation and consultation with the locals. From the remaining 109 grid cells, we randomly selected 78 (71%) for sampling, out of which 4 cells could not be surveyed due to difficult topographic conditions. Thus, the survey was carried out in 74 (68%) grid cells. The time spent to survey each of the grid cells ranged from 3 to 4.5 h and the surveys were carried out between 10 a.m. and 4 p.m.

### 2.3 Field data collection

The principal requirement of occupancy studies is the detection histories of the target species through the use of either temporal or spatial replicates (Kendall & White, 2009; Mackenzie et al., 2002). Performing temporally replicated surveys over a large area, however, often becomes unfeasible because of the associated logistical requirements (Hines et al., 2010). Due to similar reasons, we opted for spatial replicates. The replicates were delineated as linear transects of 400 m length that were arranged consecutively and were placed along the substrates that maximized detectability of indirect signs of species (Hines et al., 2010; Karanth et al., 2011). Substrates such as forest roads and trails provide an important pathway for sloth bears to travel within their habitat, thus increasing the likelihood of sign detections (Puri et al., 2015; Srivathsa et al., 2018). But, similar substrates were less available in our study area. Thus, we concentrated our survey efforts on sandy riverbeds (78%, *n* = 355,400 m transects). In the Churia habitat similar to ours, sandy riverbeds are widespread and can be instrumental in recording indirect signs, especially tracks of large carnivores (Harihar & Pandav, 2012). The remaining transects were placed along forest trails (20%, *n* = 91) and ridgelines (2%, *n* = 10). Though transects were delineated before the field surveys to ensure uniformity in spatial coverage across the grid cell, we acknowledge that in occasional cases, actual survey efforts were less than that we had expected, mostly in sites having rough and fragile terrain that had little to no availability of suitable survey routes. Hence, survey efforts varied between the grid cells both as a function of proportion of habitat available (denoted by the % of grid cell within Trijuga forest boundary) and prevailing topography in the cell (Puri et al., 2015; Wibisono et al., 2011). The number of transects ranged from 4 (in grids having difficult terrain conditions or relatively less % area within forest boundary) to 10 (in grids having accessible terrain conditions and complete area within forest boundary) and had an average of 6.16 transects/grid cell (Supplementary Material). Within the 400 m long transects, we collected sloth bear detection/nondetection data and field‐based covariates data at each 100 m segment. Only the first detected and clearly identified sign at the 100 m segment was noted as “1” indicating detection and as “0” for nondetection (Karanth et al., 2011; Mackenzie et al., 2002). Detection histories were later constructed by aggregating the segment‐level detection/nondetection data to the 400 m transects (Supplementary Material), whereas the values of field‐based covariates were all averaged at the grid cell‐level to form site covariates.

For this study, we included the indirect signs of sloth bears in the form of pugmarks, scats, and excavated holes on termite mounds and ground (Garshelis, Joshi and Smith, 1999; Puri et al., 2015; Srivathsa et al., 2018). A team of 3–5 surveyors actively looked for these signs at the 400‐m transects. The surveyors were sufficiently familiarized with sloth bear signs and survey protocols through pilot training surveys prior to the actual field surveys. We only considered relatively fresh and unambiguously identified signs for the analysis to reduce biasness that could arise from sign degradation and false‐positive detections (Miller et al., 2011; Rhodes et al., 2011). Due to the possibility of misidentifying the ground holes by sloth bears with that of other species, such as wild boars, we only consigned holes with ≥30 cm depth (Garshelis, Joshi and Smith, 1999) and containing secondary identification features (e.g., claw marks or pugmarks) to sloth bears. We carried out the field surveys in the post‐monsoon season between 2 October and 3 December 2020 as an effort of minimizing the variation in sign detection process due to rainfall (Harihar & Pandav, 2012; Karanth et al., 2011).

### 2.4 Covariates selection

We reviewed available literature on sloth bear ecology and devised covariates that seemed important in influencing the spatial pattern of sloth bears at the Trijuga forest (Table 1). Sloth bears are inclined toward myrmecophagy (Palei et al., 2014; Rather et al., 2020) and studies from Nepal show their greater reliance on termites for food (Joshi et al., 1997; Khanal & Thapa, 2015). Sloth bears prefer areas with heterogeneous terrain and proximity to water sources for different purposes such as resting, denning, and feeding (Akhtar et al., 2004; Ghimire & Thapa, 2015; Puri et al., 2015). On the other hand, sloth bears tend to avoid or react aggressively during human encounters (Ratnayeke et al., 2014; Sharp et al., 2020) and are sensitive to overharvesting of forest products, overgrazing, poaching, and minerals extraction, especially outside the PAs (Bargali et al., 2004; Dharaiya et al., 2016; Garshelis et al., 1999). Taking into account the available information, we hypothesized that the availability of termites and a high degree of terrain heterogeneity would positively influence the site use intensity of sloth bears. Similarly, we predicted the site use probability to be negatively influenced by the human disturbance factors and larger distances from the water sources. Detectability was also modeled as a function of the same site‐level covariates because of their potential in exerting fluctuations on species abundance (Royle & Nichols, 2003). This approach was also helpful in minimizing the number of parameters to be estimated during the analysis (Jathanna et al., 2015).

**TABLE 1:** Covariates devised to test their influence on the habitat use of sloth bears at the Trijuga forest, their predicted direction of influence, and the descriptive statistics of the numerical covariates at all the sampling sites (*n* = 74) and at sites where sloth bear signs were detected (*n* = 22)

Covariate	Predicted direction of influence	All sampling sites		Detection sites	
		Mean	*SE*	Mean	*SE*
Terrain ruggedness index (TRI)	Positive	100.67	3.58	99.96	7.78
Distance to the nearest water source (DW) (m)	Negative	1300	100	1029.37	112.91
Human disturbance index (HDI)	Negative	0.49	0.02	0.39	0.04
Termite mound presence (TMP)	Positive	–	–	–	–

We used various methods to note or quantify the devised covariates. Because of the difficulty in detecting underground colonies of termites, we only considered the aboveground mound‐building termites for this study. We carried out extensive searches, often deviating from the predefined transects at each grid cell, to note the presence/absence and count the number of termite mounds. Topographic heterogeneity was measured using the Terrain Ruggedness Index (TRI) developed by Riley et al. (1999) by using the Shuttle Radar Topographic Mission (SRTM) Digital Elevation Model (DEM) data with 90‐m resolution (downloaded from https://srtm.csi.cgiar.org/). The average value of TRI for each grid cell was used for the analysis (Thapa et al., 2019). We georeferenced the majority of the perennial water sources during the field surveys with the help of local field assistants. A few water bodies that we failed to locate during the surveys were digitized using Google Earth Imagery. We calculated the distance from the centroid of the grid cells to the nearest water sources using the Euclidean distance tool in ArcGIS 10.4.

Likewise, we obtained an overview of the potential anthropogenic threats to sloth bears in the study area through interactions with the locals and forest officers. These interactions revealed six major threats *viz*. human‐caused mortality (for retaliation, self‐defense, and presumably poaching), human‐induced forest fires, vegetation disturbances (logging, cutting, and looping), livestock grazing, vehicular disturbances (mainly tractors for transporting riverbed minerals, firewood, and timber), and direct human presence in bear habitat. However, bear killing was found to happen rarely and forest fires mostly occurred during the summer. Hence, we were unable to document the evidences of these threats during the course of this study. We incorporated the remaining four categories of threats to the framework of Barber‐Meyer et al. (2013) with necessary modifications for quantifying human disturbances (Thapa & Kelly, 2017; Thapa et al., 2021). We recorded the evidence of livestock and their signs (L), human presence (HP), vehicular disturbance (VeD), and vegetation disturbances (VD) at each 100 m segment. Due to the lack of published information regarding the degree of influence of these threats to sloth bears, we assigned equal weights (0.25) to each category and calculated human disturbance index at each segment as HDI = (L*0.25) + (HP*0.25) + (VeD*0.25) + (VD*0.25). We averaged the obtained value of HDI to the grid cell‐level.

### 2.5 Data analysis

We performed the single‐season occupancy analysis in program PRESENCE 13.10 (Hines, 2006). Akaike Information Criterion corrected for small sample size (AICc) was used for model comparison and selection of the best models that fit our data (Burnham & Anderson, 2002). We adopted a three‐step modeling approach to model the parameters of our interest (Karanth et al., 2011; Srivathsa et al., 2018). In the first step, we compared the standard occupancy model (Mackenzie et al., 2002) with the model accounting for correlated detections along the spatial replicates (Hines et al., 2010). We initially predicted our data to follow the correlated detections model because of the potential spatial dependence in sign detection events along the consecutive spatial replicates. After identifying the most suitable model for our data, we modeled the detection parameter either in a constant form or as a function of individual covariates (Thapa et al., 2019). The occupancy parameter at this step was kept in the most parameterized form. Finally, occupancy was modeled by fixing the covariate structure for detection probability from the top‐ranked model in the previous step (Jathanna et al., 2015; Karanth et al., 2011; Puri et al., 2015; Srivathsa et al., 2018). We used either a single or additive combination of the covariates for investigating their influence on habitat use. Models with ΔAICc < 2 were considered as competing models and the final estimates of site use probability and detectability were calculated by model averaging the competing models (Burnham & Anderson, 2002).

We computed β estimates of the covariates to understand the magnitude and direction (positive or negative) of their influence on the site use and detection probability. All the continuous covariates were normalized and checked for collinearity using the Spearman's rank correlation coefficient (*r*_s_) before the occupancy analysis. The categorical covariate indicating the presence or absence of termite mounds was coded as a binary variable represented by 1 or 0, respectively (MacKenzie et al., 2006). None of the numerical covariates were strongly correlated (all *r*_s_ < |0.5|), which enabled us to try covariate combinations without restrictions. The most parameterized model was tested for over‐dispersion by calculating c‐hat using a parametric bootstrap approach with 1000 iterations in program PRESENCE 13.10. The obtained value of c‐hat (0.20) indicated no over‐dispersion in the data (MacKenzie & Bailey, 2004). We incorporated the model‐averaged estimates from our study to ArcGIS 10.4 and prepared a predicted habitat use map of sloth bears in the Trijuga forest.

## 3 RESULTS

We carried out 182.4 km of transect walk and recorded 59 fresh signs of sloth bears. The signs were recorded in 22 grid cells that estimated naïve site use probability of 0.297. Pugmarks were the most abundantly encountered signs (54%, *n* = 32) compared to dugout holes on mounds and ground (24%, *n* = 14) and scats (22%, *n* = 13). Sign detections occurred in sites that were close to the georeferenced water sources and had less human disturbances in comparison to the sampling sites as a whole (Table 1). We documented the presence of termite mounds in 25 grid cells that had an average of 4.37 (*SE* = 0.61) mounds/ha. The majority of obtained signs were in mixed deciduous forests (76%, *n* = 45) followed by *Shorea robusta* forest (24%, *n* = 14), and no signs were detected in the riverine forests.

### 3.1 Modeling detection and habitat use probability

Contrary to our expectations, the standard model assuming independence among the detection events better fitted our data (AICc Weight = 0.69). Though the correlated detection model was also found to be competing (AICc = 1.59), its model weight was relatively low (AICc Weight = 0.31). The probability of replicate‐level presence depending on the nondetection or detection of sloth bear signs in the previous replicate [θ^0^(*SE*) = 0.97 (0.30) and θ^1^(*SE*) = 0.34 (0.17), respectively] also did not show the evidence of spatial autocorrelation among sign detections. Thereafter, we used the standard occupancy model for the analysis of our data.

**FIGURE 2:**
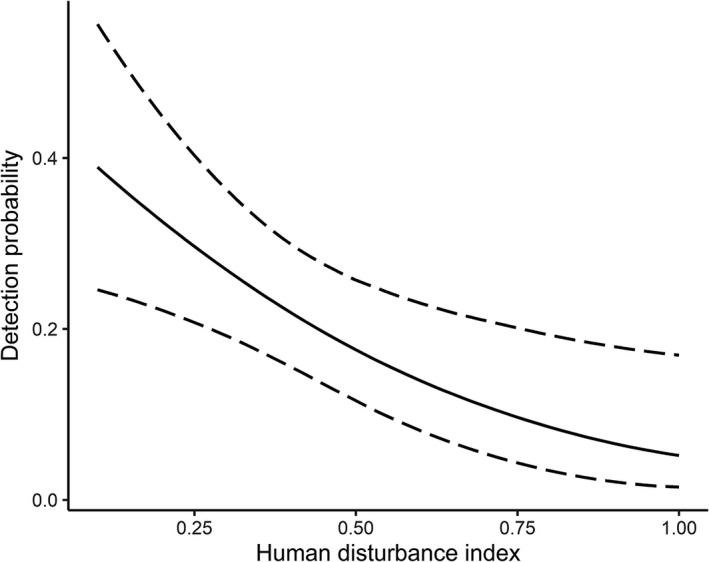
Relationship between human disturbance index (HDI) and detection probability of sloth bear signs in the Trijuga forest. The dashed lines represent the 95% confidence intervals of the detection probability

We fitted 5 regression models for detection probability, including the model with constant detection *p*(.) (Table 2). The model with human disturbance index (HDI) as a covariate for detection probability emerged at the top (AICc Weight = 0.904). The second‐best model was that with constant detection probability (ΔAICc = 5.58), but it received a very small model weight (AICc Weight = 0.05). HDI had a significant negative influence on the detection probability of sloth bear signs (β_HDI_ = −0.602, 95% CI = −1.016 to −0.188, Figure 2). Detection probability (*p* ± *SE*) ranged from 0.051 ± 0.032 in grids with high human disturbances to 0.389 ± 0.081 in grids with the least disturbances. The model averaged detection probability (*p* ± *SE*) was estimated to be 0.195 ± 0.003.

**TABLE 2:** Comparison of different models to identify the covariates influencing the detection probability of sloth bear signs in the Trijuga forest using global model ψ(TMP + DW + TRI) for occupancy

Model	AICc	ΔAICc	AICc weight	Model Likelihood	*K*	Deviance
*ψ*(Global), *p*(HDI)	245.96	0	0.904	1	6	233.06
*ψ*(Global), *p*(.)	251.84	5.88	0.0478	0.0529	5	241.2
*ψ*(Global), *p*(DW)	253.96	8	0.0166	0.0183	6	241.06
*ψ*(Global), *p*(TRI)	254.03	8.07	0.016	0.0177	6	241.13
*ψ*(Global), *p*(TMP)	254.07	8.11	0.0157	0.0173	6	241.17

Abbreviations: DW, Distance to the nearest water source; HDI, Human disturbance index; *K*, Number of parameters; TMP, Termite mound presence; TRI, Terrain ruggedness index.

For habitat use analysis, we constructed 11 regression models by fixing the HDI as a covariate for detectability (Table 3). We tried to keep the model parameters low by not including more than 2 covariates for *ψ*, except for the global model. All the fitted models received better support for the data compared to the constant model *ψ*(.) *p*(.) (ΔAICc = 17.66 for the constant model). Most support for the data was garnered by the model where *ψ* varied as an additive function of TMP and DW. The second‐ranked model had a similar covariate structure with the addition of TRI (ΔAICc = 0.58). Estimated habitat use probability (*ψ* ± *SE*) increased from 0.371 ± 0.071 in model with no covariates to 0.432 ± 0.039 in the model‐averaged estimates using the competing models. Based on the summed AICc weights of each variable, we found TMP to be the most important predictor of sloth bear habitat use (Summed AICc weight = 0.996). DW was the next important predictor (0.847), whereas TRI received less support (0.392) and HDI had relatively negligible influence (0.052) in predicting the habitat use.

**TABLE 3:** Comparison of different models to identify the covariates influencing the habitat use probability of sloth bears in the Trijuga forest using the spatially replicated sign surveys

Model	AICc	ΔAICc	AICc weight	Model Likelihood	*K*	Deviance
*ψ*(DW + TMP), *p*(HDI)	245.63	0	0.4834	1	5	234.82
*ψ*(TMP + DW + TRI), *p*(HDI)	246.21	0.58	0.3617	0.7483	6	233.06
*ψ*(TMP), *p*(HDI)	249.47	3.84	0.0709	0.1466	4	240.94
*ψ*(HDI + TMP), *p*(HDI)	250.14	4.51	0.0507	0.1049	5	239.33
*ψ*(TRI + TMP), *p*(HDI)	251.21	5.58	0.0297	0.0614	5	240.4
*ψ*(HDI), *p*(HDI)	258.12	12.49	0.0009	0.0019	4	249.59
*ψ*(DW), *p*(HDI)	258.21	12.58	0.0009	0.0019	4	249.68
*ψ*(DW + HDI), *p*(HDI)	258.59	12.96	0.0007	0.0015	5	247.78
*ψ*(TRI + HDI), *p*(HDI)	260.18	14.55	0.0003	0.0007	5	249.37
*ψ*(TRI), *p*(HDI)	260.31	14.68	0.0003	0.0006	4	251.78
*ψ*(TRI + DW), *p*(HDI)	260.46	14.83	0.0003	0.0006	5	249.65

Abbreviations: DW, Distance to the nearest water source; HDI, Human disturbance index; *K*, Number of parameters; TMP, Termite mound presence; TRI, Terrain ruggedness index.

**FIGURE 3:**
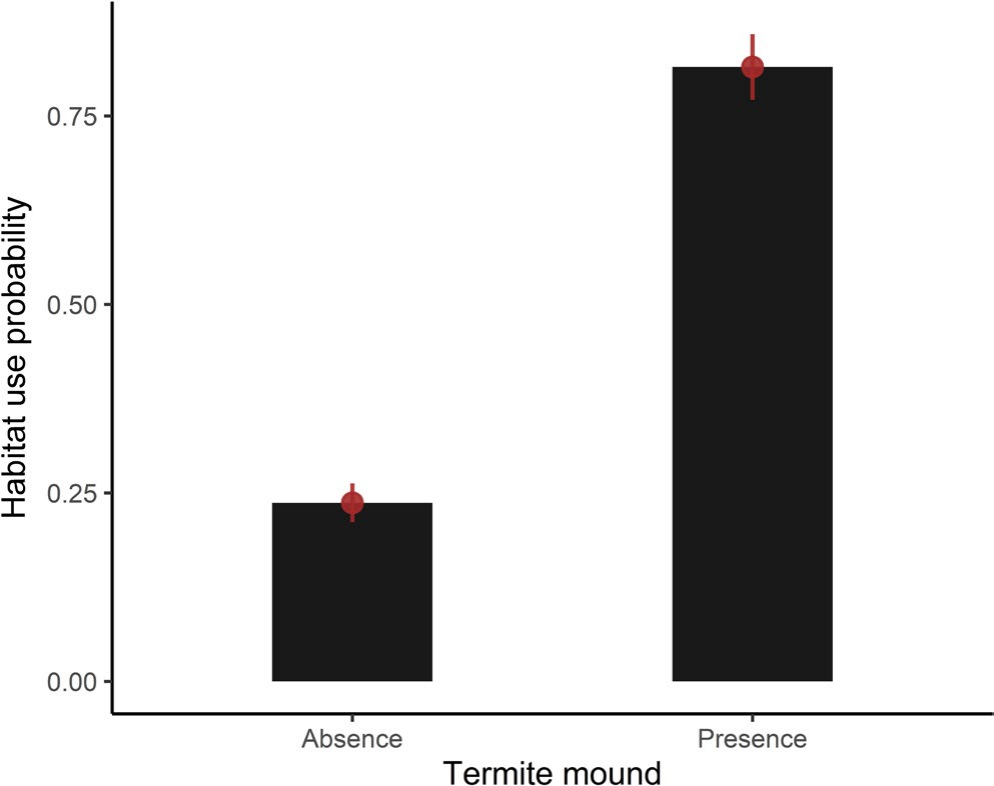
Relationship between the presence or absence of termite mounds and the habitat use probability of sloth bears in the Trijuga forest. The error bars represent the 95% confidence intervals of the habitat use probability

**FIGURE 4:**
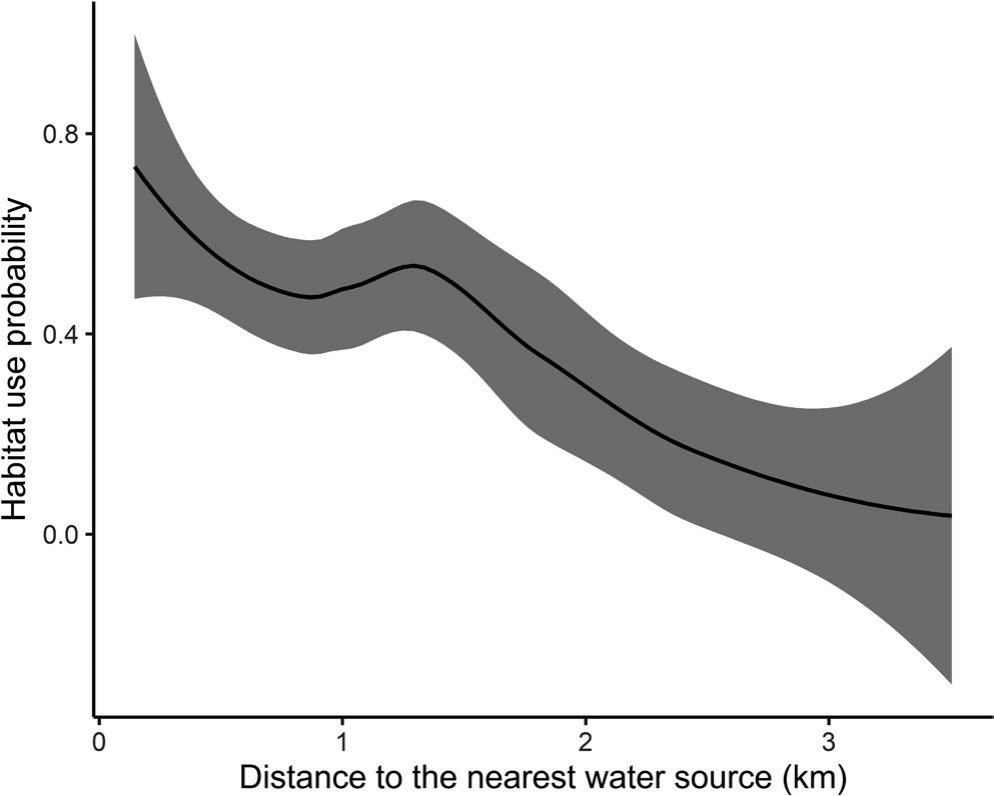
Relationship between the distance to the nearest water source (DW) and the habitat use probability of sloth bears in the Trijuga forest. The shaded area represents the 95% confidence intervals of the habitat use probability

The estimated β coefficients indicated that TMP had a strong positive influence on the habitat use probability of sloth bears (Figure 3). DW had a negative influence, indicating lower habitat use probability in sites with larger distances from the water sources (Figure 4). The 95% CIs did not overlap 0 for these covariates (β_TMP_ = 3.562, 95% CI = 0.817–6.308; β_DW_ = −1.456, 95% CI = −2.902 to −0.011). As hypothesized, TRI had a positive influence and HDI had a negative influence on the site use probability though the 95% CIs for both the covariates overlapped zero (β_TRI_ = 0.330, 95% CI = −0.598 to 1.257; β_HDI_ = −0.584, 95% CI = −1.439 to 0.272).

## 4 DISCUSSIONS

This is the first study investigating the habitat use correlates of sloth bears in the Churia habitat outside the protected areas of Nepal. Our findings shed light on the major factors influencing the site use pattern of sloth bears in this part of their range, and have provided baseline for evaluation of future trends in the site use with respect to the changes in given covariates. Furthermore, our study highlights the importance of considering the fine‐scale ecological and anthropogenic factors in predicting the sloth‐bear habitat relationships across their range in the Churia habitat of Nepal, and more specifically in the unprotected areas.

Space use patterns of large mammals, be it herbivore, carnivore, or omnivore, is most often determined by the availability and distribution of feeding resources (Barber‐Meyer et al., 2013; Dupke et al., 2017; Karanth et al., 2011; Kozakai et al., 2011). Our findings showed consistency with this hypothesis by demonstrating a strong positive influence of termite mound's presence on the habitat use by sloth bears in the Trijuga forest. Termites constitute an important part of sloth bears’ diet across much of their range, but their significance becomes more prevalent during the period of low fruit availability in the wild (Bargali et al., 2004; Joshi et al., 1997; Ratnayeke et al., 2007). Because we conducted this study after the fruiting season (May–August) of most plants in the lowlands of Nepal (Joshi et al., 1997), the sloth bears could have exhibited strong dependency on termites for diet. Similar to our results, Das et al. (2014) has reported the important function of termite mounds in the spatial pattern of sloth bears inhabiting the semi‐arid region of northeastern Karnataka in India when fruiting resources were less available. Furthermore, it has been reported that grassland habitat provides sloth bears and other bear species (e.g., Asiatic black bears *Ursus thibetanus*) important feeding ground by sheltering high density of underground termites and ants (Joshi et al., 1997; Yamazaki et al., 2012). Due to the lack of natural grassland habitat and the associated food resources in the Trijuga forest, the sloth bears could have been more reliant on the aboveground mound‐building termites for food. Nonetheless, our observation of some excavated ground holes indicates that they feed on the underground colonies of termites and ants whenever available.

Besides, it is evident from secondary data (Shah et al., 2018) and our observation that there exists some degree of competition between humans and sloth bears for the plant‐based food resources in the Trijuga forest. This kind of competition leads to the lack of food materials for sloth bears, thereby forcing them to look for anthropogenic food sources around human settlements or farmlands (Bargali et al., 2004; Prajapati et al., 2021; Rajpurohit & Krausman, 2006). However, throughout the Trijuga forest, we rarely documented the incidents of crop‐raiding by sloth bears and human attacks was the prime type of conflict reported (Pokharel & Aryal, 2020). This further points out to the myrmecophagous diet of sloth bears in this region. Limited dependency on anthropogenic food sources by sloth bear populations exhibiting strong myrmecophagy has been reported by other studies, even in areas with some level of overlap between humans and bears for the plant‐based feeding resources (Joshi et al., 1995; Rather et al., 2020; Ratnayeke et al., 2007). However, since sloth bears are known for their ability to adjust to changing food and habitat conditions (Joshi et al., 1997; Laurie & Seidensticker, 1977), it is unwise to make inferences on the feeding behavior of sloth bears without reliable supporting data. Thus, we believe that the obtained finding has opened up new avenues for further research on the feeding behavior of sloth bears in this and other parts of the Churia region in Nepal. It is essential to conduct multi‐season occupancy studies (fruiting and nonfruiting seasons) incorporating the influence of both termites and fruits together with research on feeding ecology in order to reliably ascertain the pattern of sloth bear's dietary resource utilization (Joshi et al., 1997; Ramesh et al., 2012).

Distance to water sources was the second most important predictor of habitat use by sloth bears in the Trijuga forest, whereby sloth bears tended to prefer sites that are near to the perennial sources of water. Preference of areas near to water bodies has been documented for sloth bears and other bear species, such as the American black bears (*Ursus americanus*) and Asiatic black bears, because of the potential of such sites in providing suitable foraging and denning habitat (Akhtar et al., 2004; Bashir et al., 2018; Benson & Chamberlain, 2007; Jain et al., 2021). For sloth bears in particular, termites are found to be abundant in moist soil conditions (Ratnayeke et al., 2007), and foraging them becomes easy in the well‐drained soft soils around water bodies (Akhtar et al., 2004). Thus, in the relatively dry Churia habitat, water could be an important limiting factor for sloth bears, as have been reported for other large mammals, including Bengal tigers (*Panthera tigris*) and gaurs (*Bos gaurus*; Thapa & Kelly, 2017). The importance of moist foraging sites should become more prevalent during the dry season when both termite mounds and ground soil become hard, impeding the bears’ ability to exploit them by digging (Joshi, Garshelis, & Smith, 1995, 1997). In the same way, sloth bears are known to make use of different physical features located nearby water sources (e.g., rocky outcrops, tree cavities, and erosion made cavities on ground), mostly for rearing cubs and resting (Akhtar et al., 2004; Baskaran et al., 2015). During the field work for this study, we observed extensive dugout holes by sloth bears in some areas having moist riverbeds and on the moist walls of narrow gullies. Likewise, all of the observed den sites (*n* = 5) that had evidences of sloth bears were in large tree cavities and rocky outcrops adjacent to small streams. Hence, it is somewhat evident that sites in proximity to water bodies provide sloth bears with appropriate feeding, denning, and resting habitat in the Churia hills, thereby increasing the probability of being used.

We predicted the positive influence of TRI and the negative influence of HDI on the habitat use by sloth bears. The results obtained were in congruence with the predicted direction of influence, even though the strength of association was weak and the 95% CIs of the estimates included zero for both the variables. High terrain heterogeneity is associated with complex topographic conditions that are less accessible to humans and also provide important denning and resting space for sloth bears (Puri et al., 2015). The weaker degree of support for TRI could be due to the small spatial scale (4 km^2^) of our study. Different studies have reported a weak influence of TRI in describing the fine‐scale species‐habitat relationships including for sloth bears (Srivathsa et al., 2018), American black bears (Gould et al., 2019) and Asiatic elephants (Thapa et al., 2019). Hence, if we would expand the size of our sample unit to include the area larger than the home range of sloth bears (e.g., Puri et al., 2015), there is a possibility that TRI could exhibit strong predictive power on the landscape‐scale occurrence pattern of sloth bears in our study area.

On the other hand, HDI had a moderate but significant effect on the detectability of bear signs, but very little support was obtained in explaining the habitat use pattern. It could be due to the widespread nature of human disturbances in the Trijuga forest, especially during daylight hours. Sloth bears might have been forced to use the disturbed habitats through the adoption of some spatiotemporal mechanisms of habitat segregation to minimize the degree of impact. Temporal ways of segregation, such as increased nocturnal activity, and spatial mechanisms, such as restriction of movement to certain areas of limited disturbances during times of high human activities, has been reported for several wildlife species, including bears, throughout the world (Carter et al., 2012; Gaynor et al., 2018; Martin et al., 2010). Typically, the sloth bears are crepuscular or nocturnal species (Ramesh et al., 2013; Yoganand et al., 2005), which might have facilitated some level of coexistence between humans and sloth bears in the Trijuga region. Yet, during sub‐adulthood and motherhood, sloth bears are more likely to remain active during the daytime to avoid the risks of predation and aggressive encounters with adult conspecifics (Joshi et al., 1999). This could have promoted human attacks by bears mostly during daylight as documented in parts of the Trijuga forest (Pokharel & Aryal, 2020).

Additionally, in our case, the design of spatial replicates should have contributed to the significant influence of HDI on the detectability. The replicates were predominantly placed on the dry riverbeds and trails (>90%), which were also frequently used by the local people, often accompanied by livestock or vehicles, to travel in the forest. This must have caused the destruction of sloth bear signs such as pugmarks and scats, thus limiting our ability to detect them during surveys. We suggest future studies be targeted in understanding the spatial as well as temporal variations in sloth bear use of habitat in response to human disturbances. The use of new technologies, such as camera traps can be instrumental in collecting data necessary for such analysis (Carter et al., 2012), while it also has the potential to minimize biasness arising from sign degradation by human activities that may occur along the spatial replicates.

## 5 CONCLUSIONS AND MANAGEMENT IMPLICATIONS

The conservation policies and practices in Nepal are largely biased toward the large mammals. Yet, species like the sloth bears have never garnered special conservation interest, and are not listed as the protected species of Nepal (Heinen & Yonzon, 1994). Our study demonstrates the significance of protecting the sloth bear populations outside the PAs, where they are under intense anthropogenic pressures and have their distribution minimally overlapped with the conservation‐focused species. Our results indicate that the fine‐scale space use patterns of sloth bears in the Trijuga forest is determined by the availability and distribution of basic ecological resources. Hence, the long‐term survival of sloth bears in this area can only be ensured given their foraging, denning, and resting habitat are maintained in good quality. In this regard, we suggest that the predictors of sloth bears’ site use identified in this study (i.e., the presence of termite mounds and the proximity to water sources) should also be applicable to other areas of their distribution in the Churia range of Nepal.

**FIGURE 5:**
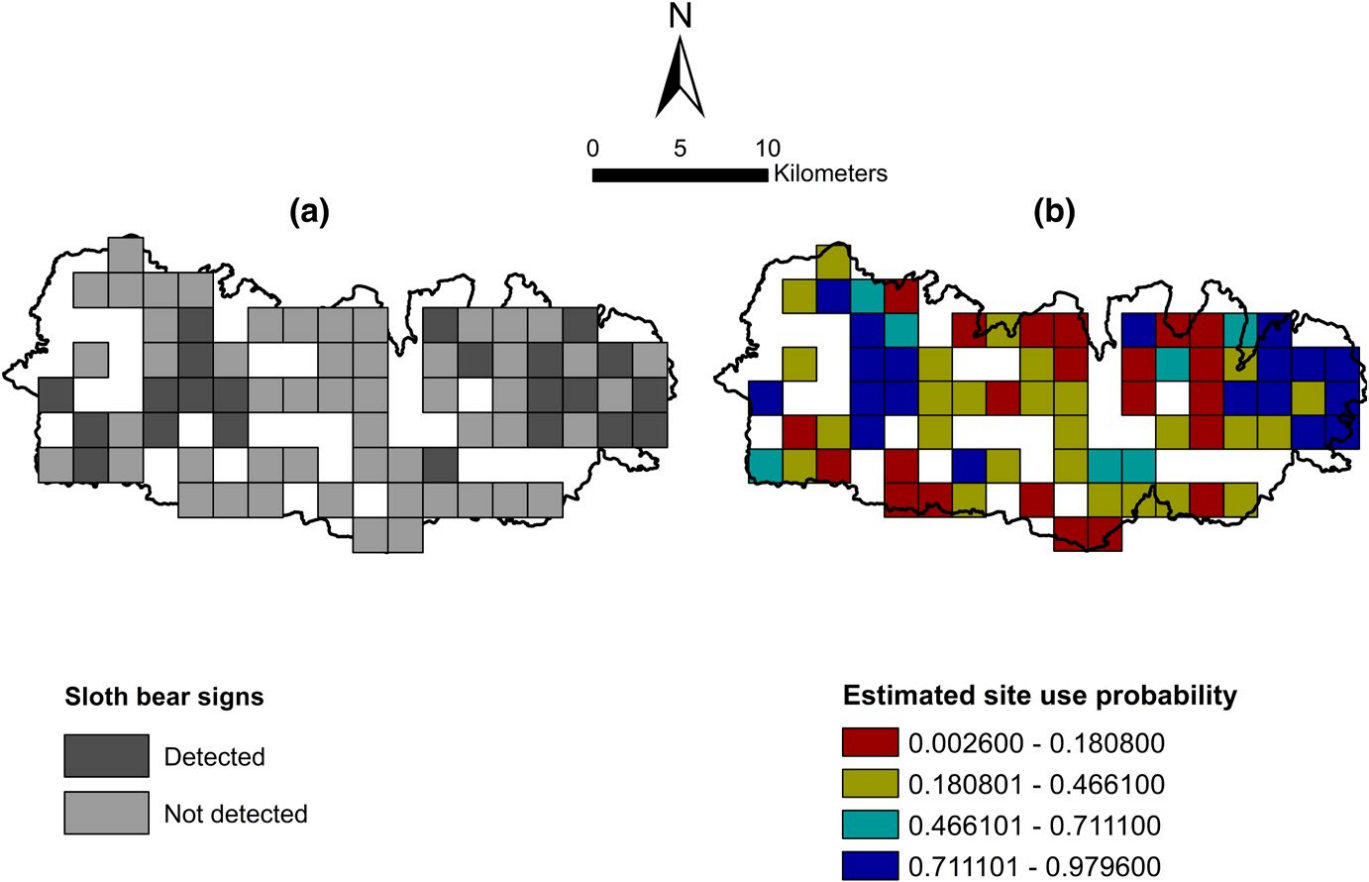
Patterns of site use by sloth bears in the Trijuga forest based on the sign surveys. (a) Naive site use (b) Estimated probabilities of site use

Moreover, given the high rate of habitat conversion, encroachment, and other anthropogenic disturbances undergoing in the Churia hills (Subedi et al., 2021), it is obvious that these basic resources are being depleted at a faster rate. Though not evident in our study, the degree of human disturbances can have a profound impact on the occurrence probability of sloth bears (Puri et al., 2015), and the major priority should be to regulate human activities in the probable areas of bear occurrence in a way that has a minimal impact on the long‐term conservation of the species. The predictive map prepared in this study has prioritized sites based on their probability of being used by sloth bears (Figure 5). For example, the sites in the eastern and west‐central part of the Trijuga forest have higher use probability. Conservation and habitat‐management interventions should, therefore, be targeted to these areas through minimization of human disturbances. Expanding similar assessments to other parts of the Churia range can help us identify major distribution hotspots of sloth bears outside the PAs of Nepal. In addition, studies like ours could act as a starting point for carrying out human‐sloth bear conflict investigation and mitigation interventions by predicting probable areas where conflicts could occur.

## CONFLICT OF INTEREST

The authors declare no conflict of interests.

## AUTHOR CONTRIBUTION

**Manoj Pokharel:** Conceptualization (lead); Data curation (lead); Formal analysis (lead); Funding acquisition (lead); Investigation (equal); Methodology (equal); Project administration (lead); Resources (equal); Validation (equal); Visualization (lead); Writing – original draft (lead); Writing – review & editing (equal). **Asmit Subba:** Investigation (equal); Methodology (equal); Resources (equal); Validation (equal); Writing – review & editing (equal). **Dipa Rai:** Investigation (equal); Resources (equal); Validation (equal); Writing – review & editing (equal). **Simrik Bhandari:** Validation (equal); Writing – original draft (supporting); Writing – review & editing (equal). **Yadav Ghimirey:** Formal analysis (supporting); Funding acquisition (supporting); Supervision (lead); Validation (equal); Writing – review & editing (equal).

## Supporting information

Supplementary Material

## Data Availability

Data associated with this manuscript can be accessed at the Dyrad data repository (https://doi.org/10.5061/dryad.1g1jwstz9).
